# Safety and Tolerability of SRX246, a Vasopressin 1a Antagonist, in Irritable Huntington’s Disease Patients—A Randomized Phase 2 Clinical Trial

**DOI:** 10.3390/jcm9113682

**Published:** 2020-11-16

**Authors:** Michael J. Brownstein, Neal G. Simon, Jeffrey D. Long, Jon Yankey, Hilda T. Maibach, Merit Cudkowicz, Christopher Coffey, Robin A. Conwit, Codrin Lungu, Karen E. Anderson, Steven M. Hersch, Dixie J. Ecklund, Eve M. Damiano, Debra E. Itzkowitz, Shifang Lu, Marianne K. Chase, Jeremy M. Shefner, Andrew McGarry, Brenda Thornell, Catherine Gladden, Michele Costigan, Padraig O’Suilleabhain, Frederick J. Marshall, Amy M. Chesire, Paul Deritis, Jamie L. Adams, Peter Hedera, Kelly Lowen, H. Diana Rosas, Amie L. Hiller, Joseph Quinn, Kellie Keith, Andrew P. Duker, Christina Gruenwald, Angela Molloy, Cara Jacob, Stewart Factor, Elaine Sperin, Danny Bega, Zsazsa R. Brown, Lauren C. Seeberger, Victor W. Sung, Melanie Benge, Sandra K. Kostyk, Allison M. Daley, Susan Perlman, Valerie Suski, Patricia Conlon, Matthew J. Barrett, Stephanie Lowenhaupt, Mark Quigg, Joel S. Perlmutter, Brenton A. Wright, Elaine Most, Guy J. Schwartz, Jessica Lamb, Rosalind S. Chuang, Carlos Singer, Karen Marder, Joyce A. Moran, John R. Singleton, Meghan Zorn, Paola V. Wall, Richard M. Dubinsky, Carolyn Gray, Carolyn Drazinic

**Affiliations:** 1 Azevan Pharmaceuticals, Inc., Bethlehem, PA 18015, USA; ngsimon@azevan.com (N.G.S.); htmaibach@gmail.com (H.T.M.); edamiano@ptd.net (E.M.D.); debrai@azevan.com (D.E.I.); sl0d@lehigh.edu (S.L.); 2 Department of Biological Sciences, Lehigh University, Bethlehem, PA 18015, USA; 3 Department of Biostatistics, University of Iowa, Iowa City, IA 52242, USA; jeffrey-long@uiowa.edu (J.D.L.); jon-yankey@uiowa.edu (J.Y.); christopher-coffey@uiowa.edu (C.C.); dixie-ecklund@uiowa.edu (D.J.E.); michele-costigan@uiowa.edu (M.C.); 4 Department of Neurology, Massachusetts General Hospital, Boston, MA 02114, USA; mcudkowicz@partners.org (M.C.); hersch@helix.mgh.harvard.edu (S.M.H.); mchase@mgh.harvard.edu (M.K.C.); bthornell@mgh.harvard.edu (B.T.); cgladden@mgh.harvard.edu (C.G.); rosas@helix.mgh.harvard.edu (H.D.R.); 5 National Institutes of Health, NINDS, Bethesda, MD 20852, USA; rc296d@nih.gov (R.A.C.); lunguci@ninds.nih.gov (C.L.); 6 Department of Neurology, Medstar Georgetown University Hospital, Washington, DC 20007, USA; kea45@georgetown.edu; 7 Voyager Therapeutics Inc., Cambridge, MA 02139, USA; 8 Barrow Neurological Institute, Phoenix, AZ 85013, USA; jeremy.shefner@dignityhealth.org; 9 Department of Neurology, College of Medicine, The University of Arizona, Phoenix, AZ 85004, USA; 10 Department of Neurology, College of Medicine, Creighton University, Phoenix, AZ 85013, USA; 11 Department of Neurology, Cooper University Hospital, Camden, NJ 08103, USA; mcgarry-andrew@cooperhealth.edu; 12 Department of Neurology, UT Southwestern Medical Center, Dallas, TX 75390, USA; padraig.osuilleabhain@utsouthwestern.edu; 13 Department of Neurology, University of Rochester Medical Center, Rochester, NY 14618, USA; fred.marshall@urmc.rochester.edu (F.J.M.); amy_chesire@urmc.rochester.edu (A.M.C.); paul_deritis@urmc.rochester.edu (P.D.); jamie_adams@urmc.rochester.edu (J.L.A.); 14 Department of Neurology, Vanderbilt University, Nashville, TN 37212, USA; phederabna@gmail.com (P.H.); kelly.lowen@vumc.org (K.L.); 15 Department of Neurology, Oregon Health and Science University, Portland, OR 97239, USA; peterami@ohsu.edu (A.L.H.); quinnj@ohsu.edu (J.Q.); keithk@ohsu.edu (K.K.); 16 Department of Neurology, University of Cincinnati, Cincinnati, OH 45219, USA; dukeraa@ucmail.uc.edu (A.P.D.); gruenwcm@vcmail.uc.edu (C.G.); angela.molloy@uc.edu (A.M.); cara.jacob@uc.edu (C.J.); 17 Department of Neurology, Emory University, Atlanta, GA 30322, USA; sfactor@emory.edu (S.F.); esperin@emory.edu (E.S.); 18 Department of Neurology, Northwestern University, Chicago, IL 60611, USA; dbega@nm.org (D.B.); zsazsa.brown@northwestern.edu (Z.B.); 19 Department of Neurology, University of Colorado Denver, Aurora, CO 80045, USA; lauren.seeberger@CUAnschutz.edu; 20 Department of Neurology, The University of Alabama at Birmingham, Birmingham, AL 35233, USA; vsung@uabmc.edu (V.W.S.); melaniebenge@uabmc.edu (M.B); 21 Department of Neurology, Ohio State University, Columbus, OH 43210, USA; sandra.kostyk@osumc.edu (S.K.K.); allison.daley@osumc.edu (A.M.D.); 22 Department of Neurology, University of California Los Angeles, Los Angeles, CA 90095, USA; sperlman@mednet.ucla.edu; 23 Department of Neurology, University of Pittsburgh Medical Center, Pittsburgh, PA 15213, USA; suskivr@upmc.edu (V.S.); pmc38@pitt.edu (P.C.); 24 Department of Neurology, Virginia Commonwealth University, Richmond, VA 23298, USA; matthew.barrett@vcuhealth.org (M.J.B.); sal3q@virgina.edu (S.L.); quigg@virginia.edu (M.Q.); 25 Department of Neurology, Washington University, Saint Louis, MO 63110, USA; perlmutterjoel@wustl.edu (J.S.P.); ecmost@wustl.edu (E.M.); 26 Department of Neurosciences, University of California San Diego, La Jolla, CA 92121, USA; b9wright@health.ucsd.edu; 27 Department of Neurology, Stony Brook University Hospital, Stony Brook, NY 11794, USA; guy.schwartz@stonybrookmedicine.edu (G.J.S.); Jessica.Lamb@stonybrookmedicine.edu (J.L.); 28 Department of Neurology, Swedish Medical Center, Seattle, WA 98122, USA; rchuangmd@gmail.com; 29 Department of Neurology, University of Miami, Miami, FL 33136, USA; csinger@med.miami.edu; 30 Department of Neurology, Columbia University, New York, NY 10032, USA; ksm1@cumc.columbia.edu (K.M.); jt617@columbia.edu (J.A.M.); 31 Clinical Neurosciences Center, University of Utah, Salt Lake City, UT 84132, USA; rob.singleton@hsc.utah.edu (J.R.S.); meghan.zorn@hsc.utah.edu (M.Z.); paola.wall@hsc.utah.edu (P.V.W.); 32 Department of Neurology, University of Kansas Medical Center, Kansas City, KS 66160, USA; rdubinsk@kumc.edu (R.M.D.); cgray1@kumc.edu (C.G.); 33 Department of Clinical Sciences, Florida State University, Tallahassee, FL 32306, USA; cdrazinic@fsu.edu

**Keywords:** Huntington’s disease, safety, tolerability, vasopressin 1a receptor antagonist

## Abstract

SRX246 is a vasopressin (AVP) 1a receptor antagonist that crosses the blood-brain barrier. It reduced impulsive aggression, fear, depression and anxiety in animal models, blocked the actions of intranasal AVP on aggression/fear circuits in an experimental medicine fMRI study and demonstrated excellent safety in Phase 1 multiple-ascending dose clinical trials. The present study was a 3-arm, multicenter, randomized, placebo-controlled, double-blind, 12-week, dose escalation study of SRX246 in early symptomatic Huntington’s disease (HD) patients with irritability. Our goal was to determine whether SRX246 was safe and well tolerated in these HD patients given its potential use for the treatment of problematic neuropsychiatric symptoms. Participants were randomized to receive placebo or to escalate to 120 mg twice daily or 160 mg twice daily doses of SRX246. Assessments included standard safety tests, the Unified Huntington’s Disease Rating Scale (UHDRS), and exploratory measures of problem behaviors. The groups had comparable demographics, features of HD and baseline irritability. Eighty-two out of 106 subjects randomized completed the trial on their assigned dose of drug. One-sided exact-method confidence interval tests were used to reject the null hypothesis of inferior tolerability or safety for each dose group vs. placebo. Apathy and suicidality were not affected by SRX246. Most adverse events in the active arms were considered unlikely to be related to SRX246. The compound was safe and well tolerated in HD patients and can be moved forward as a candidate to treat irritability and aggression.

## 1. Introduction

Huntington’s disease (HD) is an inherited disorder resulting from expansion of a trinucleotide (CAG, cytosine/adenine/guanine) repeat that encodes a polyglutamine tract in the huntingtin gene. How the protein that results from expression of the mutant gene gradually damages neurons is not fully understood but over time cellular losses result in defective motor control, involuntary movements (e.g., chorea), cognitive decline and behavioral problems [1]. For the most common CAG repeat expansions (41 to 43), symptoms typically begin between the fourth or fifth decade of life but may occur much earlier or later. Psychiatric symptoms, including irritability and aggression, are commonly seen in HD and are among the most distressing consequences for patients and their family members [1]. Such symptoms, which are reported in 40%–70% of HD patients, have adverse effects on daily life and often result in institutionalization. While potential disease modifying therapies may alter the course of the disease, they may not eliminate its symptoms. Consequently, symptomatic treatments will very likely continue to be needed. 

SRX246 is a first-in-class, highly selective, highly specific vasopressin 1a (V1a) receptor antagonist that crosses the blood-brain barrier following oral administration. The molecule exhibits high affinity and high selectivity for its target receptor, is well-tolerated and has excellent pharmacokinetics [2,3]. SRX246 has a strong safety profile and no effect on water balance because the antidiuretic action of arginine vasopressin (AVP) is mediated by V2 receptors in the kidney, not V1a receptors. Preclinical pharmacology studies demonstrated that SRX246 has significant central nervous system (CNS) effects in models including aggression, depression, anxiety and fear [2,4,5,6]. In an experimental medicine fMRI study in healthy volunteers, the compound significantly attenuated the effect of intranasal AVP in brain circuits known to modulate emotional responses to stimuli that elicit aggression/fear [7]. Together, these findings suggested that SRX246 has potential as a novel therapeutic agent for major neuropsychiatric symptoms seen in HD patients. As a step in the development of the drug for this purpose, we tested its tolerability and safety in HD patients given oral doses as high as 160 mg twice daily. 

## 2. Experimental Section

This was a 3-arm, multicenter, randomized, placebo-controlled, double-blind, 12-week, dose-escalation study. Subjects were required to have a study partner to assist with participation and provide observer-reported outcomes. Both the patient and the study partner provided written informed consent. The subjects had to be 18 years old or older and were required to have motor, cognitive or behavioral features of HD; a confirmatory family history of HD or a CAG repeat expansion >36; a Total Functional Capacity (TFC) score on the UHDRS of 5–13; evidence of irritability—specifically, a score of 2 or more on the severity measure of either the UHDRS Irritability question, 30b or Aggression question, 31b. Women of childbearing potential had to have a negative pregnancy test and must have been using adequate contraception during the study. Men must have agreed to use contraception. Subjects had to be able to swallow the drug capsules and have sufficient English skills to complete all required assessments without assistance of an interpreter. Subjects could not have behavioral problems, systemic illnesses or disabilities that could lead to difficulty complying with the protocol; a history of alcohol or substance abuse in the 2 years preceding the study; or active suicidal ideation. Since it would not be ethical to require patients who might benefit from prescription or over the counter medications to stop taking them, such people were allowed to continue to do so. Ninety-four percent of subjects used such medications with some 50% of the participants taking antidepressants. Key baseline clinical characteristics are shown in Table 1. 

**Figure 1:**
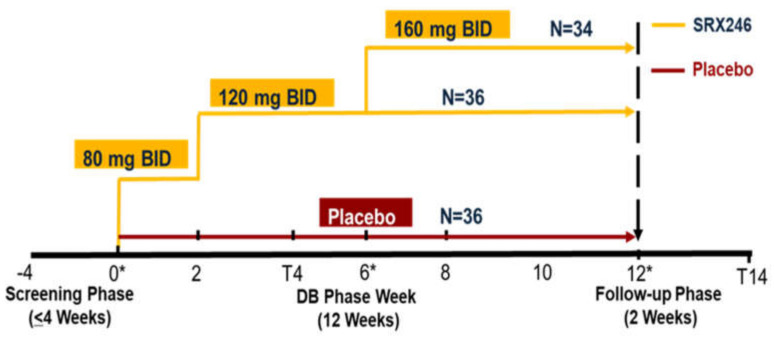
The STAIR trial (Safety, Tolerability, and Activity of SRX246 in Irritable Subjects with Huntington’s Disease) graphic illustrates the dosing and dose escalation schedule in the study. The treatment for each subject was assigned by a randomized code. A block randomization scheme was used to ensure approximately even distribution of subjects in treatment groups at each site (1:1:1 allocation). All subjects in the active arms were given 80 mg BID SRX246 for two weeks to provide an initial assessment of safety risks. Active arm subjects all escalated to 120 mg BID at week 2 and at week 6, half were escalated to 160 mg BID. The final sample size (*N*) randomized to each dose group is shown.

There was random assignment to three treatment arms: 120 mg SRX246, 160 mg SRX246 and placebo. All capsules were identical in appearance and subjects took one capsule twice a day (BID) for a total of 12 weeks (Figure 1). A randomized permuted block design was used (blocks of 6 and 9) to ensure approximately even distribution in treatment groups at each site, with equal arm allocation (1:1:1). Statisticians at the Data Coordinating Center (DCC) generated and validated a randomized treatment schedule that was incorporated into the electronic data capture (EDC) system. Only the DCC statisticians and data managers had access to unblinded treatment assignments; all other personnel were blinded throughout the study.

In the first 2 weeks, patients in the active arms were given 80 mg of SRX246 twice daily (BID). There were no tolerability or safety issues and patients were then given 120 mg of drug BID for 4 weeks. Assuming they had again tolerated the compound and there were no significant safety issues, they were given either 120 mg or 160 mg BID for an additional 6 weeks. The BID dosing regimen was based on PK results obtained in a Phase 1 Multiple Ascending Dose trial (NCT01088932). Study visits occurred at baseline; 2, 4, 6, 8, 10 and 12 weeks either in person or by telephone. A schedule of visits and assessments is provided in the Supplementary Materials, Table S1. 

Patients who could not tolerate their dose of drug (or placebo) could have the dose reduced without compromising the blinding. Medication adherence was tracked by counting capsules in bottles brought to clinic visits and with spot checks of blood levels. The study was approved by a central Institutional Review Board (IRB; Massachusetts General Brigham) as part of the NeuroNext program and secondarily by IRBs at the 22 participating sites. It was registered at www.clinicaltrials.gov, identifier NCT2507284. The roles of the site Principal Investigators (PIs), PI, IRB Independent Medical Monitor and members of the Data Safety Monitoring Board (DSMB) in guaranteeing the safety of trial participants are described in an NINDS Guidelines document: https://www.ninds.nih.gov/Funding/Apply-Funding/Application-Support-Library/NINDS-Guidelines-Data-and-Safety-Monitoring. There were no explicit stopping rules in the protocol. Site PIs determined whether reported events were classified as AEs or SAEs. Guidance on the latter was explicitly provided in the protocol. 

### Statistical Analysis

The primary objective of the trial was to assess tolerability, which was defined as the proportion of enrolled subjects completing the study on their assigned dose. Completion could occur despite temporary drug interruptions, protocol deviations and so forth. A “non-completer” was a study participant who withdrew from the study or completed it on a dose lower than their assigned one. Thus, the tolerability variable was a dichotomous indicator (yes/no) of whether participants completed the study at their assigned dose (SRX246 or placebo).

The primary analysis was performed consistent with the intent-to-treat principle. Each treatment group was compared to the placebo control group using a test of non-inferiority [8]. Non-inferiority was used because (1) it allows for powering an analysis based on the rejection of the null hypothesis that the population proportion difference is greater than or equal to a margin value (rather than non-rejection); (2) the margin value of δ = 0.50 was based on previous research by the Huntington Study Group [9,10,11,12]; and (3) the approach is considered acceptable because of the paucity of effective treatments for HD [9].

The non-inferiority test is one-tailed, as H_0_ and H_A_ are directional hypotheses, namely H_0_: π_placebo_ − π_120 mg_ ≥ δ and H_A_: π_placebo_ − π_120 mg_ < δ for the placebo vs. 120 mg group, with similar hypotheses for the 160 mg arm. The statistical test of *H*_0_ was performed using a one-sided confidence interval (CI) for the difference of the proportions based on exact methods. *H*_0_ is rejected if the upper bound of the CI is less than the margin value of 0.50. Though an exact CI has a pre-set minimum coverage probability, its length can be too long, which means the associated inverted test has low statistical power [13]. To address this issue, a computer-intensive method was used to select the shortest one-sided CI for testing [14]. The method was applied using the BinomCI function of the ExactCIdiff package [15] of the R statistical computing program.

The required sample size for the primary aim was computed based on the asymptotic test for a difference of proportions [8] between the placebo group and one treatment group. A type I error rate of α = 0.05 / 2 = 0.025 was used to allow for two comparisons (each dose level vs. placebo), with power set at 80%, meaning a one-sided CI of 97.5% was computed. The results of the power analysis (see Supplementary Materials, Figure S1) indicated that a total sample size of *N* = 108 would provide high statistical power for a reasonable range of anticipated group differences and other parameter settings.

The secondary aim of the study was to assess safety. The safety analysis focused on the proportion of adverse events (AEs) and serious adverse events (SAEs) among the groups. An AE was defined as any untoward medical occurrence associated with the use of a drug in humans, whether or not considered drug related.

To be consistent with the primary aim, the proportion of AEs was also tested with a one-sided non-inferiority test, using *α* = 0.025 and *δ* = 0.50. However, because more AEs (and SAEs) were expected for the SRX246 groups, the hypotheses were altered such that H_0_: π_120 mg_ − π_placebo_ ≥ δ and H_A_: π_120 mg_ − π_placebo_ < δ for the 120 mg vs. placebo group, with similar hypotheses for the other arm. In addition, the study was not powered for the secondary aims and it was unknown beforehand if the sample size provided enough statistical power. As part of the safety analysis, we looked at the relative risk of suicidality and apathy in the different treatment groups because these are increased by drugs that are commonly used to treat HD. Suicidality was compared across treatments based on Columbia Suicide Severity Rating Scale C-SSRS scores and apathy was measured using the apathy subscale of the Problem Behavior Assessment-short form.

Exploratory relative risk (RR) analyses were conducted on specific AEs (see Table 5b). The RR point estimate was defined as the proportion of AEs in one group (e.g., 120 mg) divided by those in another group (e.g., placebo). A 95% CI was computed for each RR without adjustment for multiple tests, as the CI was used to indicate variability in the estimate rather than for purposes of inference. (A power analysis was not performed for the RR analyses).

## 3. Results

### 3.1. Recruiting

The first subject entered the trial in May, 2016 and the last completed it in September, 2018. We screened 125 candidates to enroll 106 subjects at 22 sites, which provided sufficient power to test for tolerability. Reasons for exclusion included low levels of irritability or aggression based on UHDRS behavioral questions 30b and 31b, low total functional capacity (TFC), concerns about compliance and alcohol or substance abuse. Seven individuals elected not to participate. The modal number of recruits/site was 4 and the range was 1 to 10.

### 3.2. Demographics

Table 1 lists the characteristics of the trial participants. Approximately equal numbers of men and women participated in the study with a mean age of ~50 years and a predominance of Caucasians.

### 3.3. Primary Aim: Tolerability

Table 2 shows the results of the tolerability analysis. The one-sided CIs of the proportion difference shows that neither contained the margin value of 0.50, indicating that neither dose of SRX246 was statistically inferior to placebo with respect to tolerability.

As shown in Table 2, there were 24 (23%) non-completers. Six subjects withdrew from the trial on their own and provided no additional data (two were members of the placebo group and 4 were in the 120 mg group). The remaining 18 subjects either withdrew without giving a reason (2) or did not withdraw but completed on a dose lower than their assigned dose (15). One patient became pregnant and was withdrawn from the study. Of those who gave a reason for withdrawing from the trial, one said that problems at home made continuing difficult, another could not remain in the study because his study partner required hospitalization and could not be replaced and the remainder reported that the study visits were too burdensome.

### 3.4. Secondary Aim: Safety

Table 3 and Table 4 list the non-inferiority test results for adverse events (AEs) and serious adverse events (SAEs), respectively. Table 3 shows that there were a relatively large number of participants reporting AEs (85 across all groups), whereas Table 4 shows that the number of SAEs was small (9 total). The null hypothesis of greater than 0.50 difference in proportions was rejected for AEs and SAEs for both treatment vs. placebo comparisons. That is, the SRX246 treatment groups were not statistically inferior to the placebo group in terms of subjects who experienced adverse events.

Two hundred AEs were reported in a total of 85 of the trial participants. Table 5a lists AEs by organ system and a full list is in Supplementary Materials, Table S2. None of the AEs were very common. Eight subjects who were treated with SRX246 complained of mild-to-moderate, transient nausea (11.4%); none who took placebo did. Six subjects (8.6%) in the active arms complained of fatigue vs none in the placebo group. Somnolence was more common in patients who took placebo, however. Four subjects in the active arms had hepatic enzyme increases that were reported as AEs. None of the increases in enzyme levels were associated with elevated bilirubin, that is, loss of liver function [16]. Finally, headaches were seen more often in the subjects who took SRX246 (6 subjects) than in those given placebo (1 subject).

There were 9 severe adverse events reported (Table 5a); four resulted in dose reductions. Of the remaining five, three occurred in subjects assigned to the 120 mg group. One subject became physically aggressive; a second found that he had a small tumor at the base of one kidney, which was removed surgically; the third subject fell in the bathroom and broke her ankle. Two SAEs occurred in subjects assigned to the 160 mg group. One subject had a physical altercation that resulted in no injuries. The other subject attempted suicide. The medical monitor determined that this was unlikely to be related to study drug. In addition, the medical monitor considered none of the SAEs as unexpected or drug-related.

### 3.5. Apathy

We looked for an increase in apathy in SRX246 treated subjects because this has a negative impact on the quality of life of both patients and caregivers. It is a side effect of many of the drugs (e.g., antipsychotics) that are given to HD patients off label to treat their behavioral problems. At baseline, the Problem Behavior Assessment-short form provided data on each subject’s apathy severity and frequency over the prior 4 weeks as reported by the study partner or patient and the investigator’s interpretation of these. The majority of subjects infrequently showed apathy at baseline, week 6 and end of treatment and the most common ratings were mild or no apathy. The treatment group means for the multiplicative Apathy factor score (severity * frequency) were not statistically different at the baseline, week 6 or week 12 visits (Table 6).

### 3.6. Suicidality

The drugs used to treat chorea in HD patients (tetrabenazine and deutetrabenazine) increase the risk of depression and suicidal thoughts and behavior. We looked for this problem in SRX246-treated subjects. The presence of suicidal ideation or behavior, as measured by C-SSRS, is shown in Table 7. There were no differences between treatment groups at Visit 1 (screening, which reflects lifetime ideation) or Visit 2 (baseline), where ideation was very low in all groups. From weeks 3–7, positive suicidal ideation or behaviors were too infrequent to support statistical comparisons [17]. Three placebo subjects and three SRX246 subjects reported suicidal ideation at one of these visits. One subject attempted suicide by taking an excess of other drugs. Immediately after doing this, the patient called for help, was taken to a hospital and recovered.

## 4. Discussion

In this 3-arm, multicenter, randomized, placebo-controlled, double-blind study, neither of the SRX246 doses given was inferior to placebo in terms of tolerability or safety.

Recruiting HD patients and study partners went well. Only 125 patients had to be screened to enroll 106 subjects but by the end of the trial, recruiting subjects became somewhat more difficult. Subjects per site ranged from 1 to 10; the modal number was 4.

The demographic profile of the participants was as expected. Most of the subjects were Caucasian despite considerable effort to recruit African Americans and members of other underrepresented groups based on advice from the NeuroNext Diversity Committee. The baseline characteristics that we selected for were well balanced across groups. Other measures (e.g., UHDRS independence scores, care giver burden scores) varied across groups but these had no effect on the tests of the primary and secondary endpoints.

Regarding the 24 subjects who failed to complete the study at their assigned dose of drug or placebo, some reported that the trial was too burdensome, while others dropped out without telling us why. A third group had their doses reduced because of adverse events or severe adverse events. The AEs were spread across multiple systems and few affected many subjects (See Table 5a). Falls were expected because they are often seen in HD patients. Fatigue, nausea, headaches, abdominal pain, upper respiratory tract infections and somnolence were also frequently reported but these are often seen in the general population and in clinical trials. While we rarely saw elevations in liver enzymes in earlier trials, there were 5 in drug treated subjects in the present study. These were not accompanied by increases in bilirubin, which is the marker for liver injury [16]. Use or abuse of prescription or over the counter medications (e.g., risperidone) may have driven most of these but the liver dysfunction that is known to be a feature of HD could have been a contributing factor as well [18,19].

The absence of a change in apathy in patients who were given SRX246 contrasts with the effects of drugs (e.g., antipsychotics, anxiolytics) that are currently are used off-label to treat neuropsychiatric symptoms including irritability and aggression in HD.

The two drugs that have been approved for treating chorea in HD, tetrabenazine and deutetrabenazine, increase the risk of depression and suicide in patients. SRX246 did not appear to do this. One patient with a history of attempted suicide made an attempt, called for assistance and recovered. In future studies, a history of suicidal attempts will be incorporated in the exclusion criteria.

The results of the STAIR study indicate that SRX246 is safe and well tolerated to move forward as a candidate for treating irritability, anger and aggression in HD. The drug may also be helpful to patients with other diseases who suffer from these symptoms.

## Figures and Tables

**Table 1:** Baseline clinical characteristics by treatment group.

Characteristic *	Variable	Placebo (*N* = 36)	120 mg (*N* = 36)	160 mg (*N* = 34)	N (% of total *N* =106)
**Gender**	Male	16 (44.44%)	18 (50.00%)	17 (50.00%)	51 (48.11%)
	Female	20 (55.56%)	18 (50.00%)	17 (50.00%)	55 (51.89%)
	Missing	0	0	0	0
**Race**	White	36 (100.0%)	36 (100.0%)	33 (97.06%)	105 (99.06%)
	Not White	0 (0.00%)	0 (0.00%)	1 (2.94%)	1 (0.94%)
	Unknown/Not Reported	0	0	0	0
**Ethnicity**	Hispanic/Latino	0 (0.00%)	2 (5.56%)	4 (11.76%)	6 (5.71%)
	Not Hispanic/Latino	35 (100.0%)	34 (94.44%)	30 (88.24%)	99 (94.29%)
	Missing	1	0	0	1
**Age**	Mean (SD)	51.7 (10.4)	51.1 (13.2)	48.9 (12.7)	50.6 (12.1)
	Min.-Max.	32 - 72	19 - 77	27 - 78	19 - 78
	Missing	0	0	0	0
**Years of Education**	1–11 years	1 (2.78%)	2 (5.56%)	0 (0.00%)	3 (2.83%)
	High school/Assoc./Tech.	18 (50.00%)	24 (66.67%)	20 (58.82%)	62 (58.49%)
	Bachelors or Higher	17 (47.22%)	10 (27.78%)	14 (41.18%)	41 (38.68%)
	Unknown/Missing	0	0	0	0
**UHDRS Irritability Severity (q30b)**	Well Controlled	1 (3%)	0 (0%)	0 (0%)	*p* = 0.82
	Questionable	0 (0%)	1 (3%)	0 (0%)	
	Definite but Mild **	8 (22%)	9 (25%)	13 (38%)	
	Moderate	22 (61%)	23 (64%)	19 (56%)	
	Severe	5 (14%)	3 (8%)	2 (6%)	
**UHDRS Aggression Severity (q31b)**	Well Controlled	8 (22%)	6 (16%)	5 (15%)	*p*= 0.80
	Verbal Threats	9 (25%)	9 (25%)	12 (36%)	
	Mild Physical **	8 (22%)	14 (39%)	9 (26%)	
	Definite Physical	9 (25%)	5 (14%)	7 (21%)	
	Severe Physical	2 (6%)	2 (6%)	1 (3%)	

* There were no statistically significant differences between treatment groups. ** These responses to the UHDRS questions at baseline were the minimum for inclusion in the study. Bold formatting used for emphasis.

**Table 2:** Results for the primary endpoint, tolerability of SRX246, using a test for non-inferiority with a threshold of 0.50. The null hypothesis is that the placebo minus treatment proportion difference is greater than (or equal to) the margin of 0.50 and the alternative hypothesis is that the difference is less than 0.50.

	Group		
	Placebo	120 mg	160 mg
Sample Size	**36**	**36**	**34**
Number of completers (%)	30 (83%)	25 (69%)	27 (79%)
One-Sided 97.5% CI of Proportion Difference		(−1, 0.35)	(−1, 0.27)
Null Hypothesis Decision		Reject	Reject

**Table 3:** Results for the secondary safety endpoint, adverse events (AEs) in study subjects, using a test for non-inferiority with a threshold of 0.50. The null hypothesis is that the treatment minus placebo proportion difference is greater than (or equal to) the margin of 0.50 and the alternative hypothesis is that the difference is less than 0.50.

	Group		
	Placebo	120 mg	160 mg
Sample Size	36	36	34
Number of subjects with AEs (%)	26 (72%)	29 (81%)	30 (88%)
One-Sided 97.5% CI of Proportion Difference		(−1, 0.28)	(−1, 0.36)
Null Hypothesis Decision		Reject	Reject

**Table 4:** Results for the secondary safety endpoint, serious adverse events (SAEs) in study subjects, using a test for non-inferiority with a threshold of 0.50. The null hypothesis is that the treatment minus placebo proportion difference is greater than (or equal to) the margin of 0.50 and the alternative hypothesis is that the difference is less than 0.50.

	Group		
	Placebo	120 mg	160 mg
Sample Size	36	36	34
Number of subjects with SAEs (%)	1 (3%)	6 (17%)	2 (6%)
One-Sided 97.5% CI of Proportion Difference		(−1, 0.03)	(−1, 0.12)
Null Hypothesis Decision		Reject	Reject

**Table:** (**a**)

				Group								
System Organ Class		Placebo			120 mg			160 mg		RR (95% CI)		
		*N* = 36			*N* = 36			*N* = 34				
	# (%) of	# of	Rate	# (%) of	# of	Rate	# (%) of	# of	Rate	Placebo vs. 120 mg	Placebo vs 160 mg	120 mg vs. 160 mg
	Subjects	Events		Subjects	Events		Subjects	Events				
Blood	0 (0.0%)	0	0.000	1 (2.8%)	1	0.009	0 (0.0%)	0	0.000	N/A	N/A	N/A
Cardiac	2 (5.6%)	2	0.018	1 (2.8%)	1	0.009	1 (2.9%)	1	0.009	0.50 (0.05, 5.27)	1.89 (0.18, 19.89)	0.94 (0.06, 14.51)
Gastrointestinal	4 (11.1%)	4	0.035	7 (19.4%)	13	0.117	9 (26.5%)	14	0.122	1.75 (0.56, 5.46)	0.42 (0.14, 1.24)	0.73 (0.31, 1.75)
General	2 (5.6%)	2	0.018	3 (8.3%)	3	0.027	5 (14.7%)	7	0.061	1.50 (0.27, 8.45)	0.38 (0.08, 1.82)	0.57 (0.15, 2.19)
Infections	9 (25.0%)	11	0.097	6 (16.7%)	7	0.063	4 (11.8%)	4	0.035	0.67 (0.26, 1.68)	2.13 (0.72, 6.26)	1.42 (0.44, 4.59)
Injury, Poisoning and Procedural Comp.	5 (13.9%)	5	0.044	4 (11.1%)	6	0.054	4 (11.8%)	7	0.061	0.80 (0.23, 2.74)	1.18 (0.35, 4.03)	0.94 (0.26, 3.48)
Investigations	6 (16.7%)	8	0.071	8 (22.2%)	8	0.072	8 (23.5%)	9	0.078	1.33 (0.51, 3.46)	0.71 (0.27, 1.83)	0.94 (0.40, 2.23)
Metabolic	1 (2.8%)	1	0.009	1 (2.8%)	1	0.009	0 (0.0%)	0	0.000	1.00 (0.07, 15.38)	N/A	N/A
Musculoskeletal	1 (2.8%)	1	0.009	5 (13.9%)	5	0.045	5 (14.7%)	6	0.052	5.00 (0.61, 40.70)	0.19 (0.02, 1.54)	0.94 (0.30, 2.98)
Neoplasms	0 (0.0%)	0	0.000	1 (2.8%)	1	0.009	1 (2.9%)	1	0.009	N/A	N/A	0.94 (0.06, 14.51)
Nervous System	8 (22.2%)	9	0.080	6 (16.7%)	7	0.063	4 (11.8%)	6	0.052	0.75 (0.29, 1.94)	1.89 (0.63, 5.70)	1.42 (0.44, 4.59)
Psychiatric	4 (11.1%)	6	0.053	8 (22.2%)	11	0.099	8 (23.5%)	9	0.078	2.00 (0.66, 6.06)	0.47 (0.16, 1.43)	0.94 (0.40, 2.23)
Renal	1 (2.8%)	1	0.009	2 (5.6%)	2	0.018	2 (5.9%)	2	0.017	2.00 (0.19, 21.09)	0.47 (0.04, 4.97)	0.94 (0.14, 6.33)
Reproductive	0 (0.0%)	0	0.000	1 (2.8%)	1	0.009	0 (0.0%)	0	0.000	N/A	N/A	N/A
Respiratory	1 (2.8%)	1	0.009	3 (8.3%)	5	0.045	4 (11.8%)	4	0.035	3.00 (0.33, 27.50)	0.24 (0.03, 2.01)	0.71 (0.17, 2.94)
Skin	1 (2.8%)	1	0.009	2 (5.6%)	2	0.018	1 (2.9%)	1	0.009	2.00 (0.19, 21.09)	0.94 (0.06, 14.51)	1.89 (0.18, 19.89)
Vascular	0 (0.0%)	0	0.000	1 (2.8%)	1	0.009	2 (5.9%)	2	0.017	N/A	N/A	0.47 (0.04, 4.97)
**Total**	**26 (72.2%)**	**52**	**0.460**	**29 (80.6%)**	**75**	**0.676**	**30 (88.2%)**	**73**	**0.635**	**1.12 (0.86, 1.44)**	**0.82 (0.65, 1.04)**	**0.91 (0.75, 1.12)**

**Table:** (**b**)

					Group							
System Organ Class		Placebo			120 mg			160 mg		RR (95% CI)		
		*N* = 36			*N* = 36			*N* = 34				
	# (%) of	# of	Rate	# (%) of	# of	Rate	# (%) of	# of	Rate	Placebo vs. 120 mg	Placebo vs. 160 mg	120 mg vs. 160 mg
	Subjects	Events		Subjects	Events		Subjects	Events				
**Cardiac**												
Angina pectoris	0 (0.0%)	0	0.000	1 (2.8%)	1	0.009	0 (0.0%)	0	0.000	N/A	N/A	N/A
**Infections**												
Staphylococcal skin infection	1 (2.8%)	1	0.009	0 (0.0%)	0	0.000	0 (0.0%)	0	0.000	N/A	N/A	N/A
**Injury, Poisoning and Procedural Comp.**												
Ankle fracture	0 (0.0%)	0	0.000	1 (2.8%)	1	0.009	0 (0.0%)	0	0.000	N/A	N/A	N/A
**Musculoskeletal**												
Muscular weakness	0 (0.0%)	0	0.000	1 (2.8%)	1	0.009	0 (0.0%)	0	0.000	N/A	N/A	N/A
**Neoplasms**												
Renal neoplasm	0 (0.0%)	0	0.000	1 (2.8%)	1	0.009	0 (0.0%)	0	0.000	N/A	N/A	N/A
**Psychiatric**												
Aggression	0 (0.0%)	0	0.000	0 (0.0%)	0	0.000	1 (2.9%)	1	0.009	N/A	N/A	N/A
Irritability	0 (0.0%)	0	0.000	1 (2.8%)	1	0.009	0 (0.0%)	0	0.000	N/A	N/A	N/A
Psychotic disorder	0 (0.0%)	0	0.000	1 (2.8%)	1	0.009	0 (0.0%)	0	0.000	N/A	N/A	N/A
Suicide attempt	0 (0.0%)	0	0.000	0 (0.0%)	0	0.000	1 (2.9%)	1	0.009	N/A	N/A	N/A
**Total**	**1 (2.8%)**	**1**	**0.009**	**6 (16.7%)**	**6**	**0.054**	**2 (5.9%)**	**2**	**0.017**	**6.00 (0.76, 47.36)**	**0.47 (0.04, 4.97)**	**2.83 (0.61, 13.09)**

Column Definitions: 1: System Organ Class of AE; 2,5,8: Number and percentage of subjects who have ever had AE; 3,6,9: Total number of AEs; includes multiple AEs of same type per subject 4,7,10: Number of AEs per 30 days; 11: Relative Risk (120 mg relative to Placebo) and 95% confidence interval; 12: Relative Risk (Placebo relative to 160 mg) and 95% confidence interval; 13: Relative Risk (120 mg relative to 160 mg) and 95% confidence interval.

**Table 6:** Problem Behavior Assessment (PBA-s) Apathy Factor by Treatment Group and Study Visit (ITT Population). Apathy behavior is rated for frequency and severity on a scale of 0–4 and the Apathy Factor is calculated as frequency * severity.

Time Point	Statistic	Placebo (*N* = 36)	SRX246 120 mg BID (*N* = 36)	SRX246 160 mg BID (*N* = 34)
Visit 2, Baseline	n	35	36	34
	Mean (SD)	3.5 (3.91)	4.0 (4.37)	3.4 (5.01)
	Median	2.0	2.0	1.0
	Min, Max 95% CI	0, 16 (2.1, 4.8)	0, 12 (2.5, 5.5)	0, 16 (1.7, 5.2)
Visit 4, Week 6	n	35	31	33
	Mean (SD)	2.9 (4.38)	2.9 (3.61)	3.4 (4.64)
	Median	1.0	2.0	1.0
	Min, Max 95% CI	0, 16 (1.4, 4.4)	0, 12 (1.6, 4.2)	0, 16 (1.7, 5.0)
Visit 7, End of Treatment	n	34	31	33
	Mean (SD)	3.0 (4.17)	3.4 (4.27)	3.8 (4.77)
	Median	1.0	2.0	2.0
	Min, Max 95% CI	0, 16 (1.5, 4.5)	0, 16 (1.8, 5.0)	0, 16 (2.1, 5.0)

**Table 7:** The number and percent of participants in each group that showed suicidal ideation at screening (Visit 1), baseline (visit 2) and end of study (visit 7). Those in the “yes” group had a score other than zero based on the responses to questions 1–5 on the C-SSRS: 1—Wish to be Dead; Category 2—Non-specific Active Suicidal Thoughts; 3—Active Suicidal Ideation with Any Methods without Intent to Act; 4—Active Suicidal Ideation with Some Intent to Act, without Specific Plan; 5—Active Suicidal Ideation with Specific Plan and Intent. There were no differences between SRX120 mg vs. placebo or SRX246 160 mg vs. placebo. There also were no differences between the SRX246 groups and placebo at visits 3–6 (not shown). * The elevated scores at Visit 1 (screening) reflect lifetime suicidal ideation and are thus substantially increased compared to assessments conducted during the course of the study (visits 2–7).

	Placebo *N* = 36	Up to 120 mg *N* = 36	Up to 160 mg *N* = 34
Visit	Yes	Yes	Yes
1 *	10 27.8%	14 38.9%	11 32.4%
2	4	3	0
	11.1%	8.3%	0.0%
7	0	1	1
	0.0%	2.8%	2.9%

## Supplementary Materials

The following are available online at https://www.mdpi.com/2077-0383/9/11/3682/s1, Figure S1: Power Analysis for the Placebo Single-Arm Sample Size; Table S1: Schedule of Visits and Assessments; Table S2: Adverse Events by System Organ Class and Treatment Group.
