# Supplementary material for: Safety and Tolerability of SRX246, a Vasopressin 1a Antagonist, in Irritable Huntington’s Disease Patients—A Randomized Phase 2 Clinical Trial
Source: J Clin Med. 2020 Nov 16;9(11):3682. doi: 10.3390/jcm9113682 (PMC7696926; doi:10.3390/jcm9113682)

Supplementary Table S1. The schedule of visits and assessments conducted from screening to the end of study visit. In addition, participants and their study partners (caregivers) answered questions in an eDiary (electronic Patient Reported Outcome; ePRO) about mood and aggressive outbursts using a web application that was developed for this study.

Week	SC	BL	2	4	6	8	10	12	F/U
Visit Window	Day -30 to 0	Day 0	Day 14±5	Day 28±5	Day 42±5	Day 56±5	Day 70±5	Day 84±5	Day 91±5
Visit	SC	BL	01	Tl	02	03	04	05	T2
**Assessment** / **Dosing (BID)**	**none**	**80 mg**	**120 mg**		**120 or 160mg**				**none**
Review Study Drug Compliance			X	X	X	X	X	X	
Assessments for Adverse Events		X	X	X	X	X	X	X	X
Assessments for ConcomitantMedicationUse		X	X	X	X	X	X	X	X
Columbia Suicide Severity Rating Scale (CSSRS)	X	X	X		X	X	X	X	
Unified Huntington Disease Rating Scale (UHDRS)	X	X			X			X	
Clinical Global Impression-Severity of Illness(CGI-S)		X							
Clinical Global Impression-Improvement(CGI-I)					X			X	
Provide Diary and Instructions		X	R	R	R	R	R	R	
Aberrant Behavior Checklist (ABC), irritability subscale		X			X			X	
Cohen-Mansfield Aggression Inventory(CMAI)		X			X			X	
ProblemBehaviorsAssessment-short form(PBA-s)		X			X			X	
Irritability Scale		X			X			X	
HD QoL		X			X			X	
Caregiver Burden Questionnaire		X			X			X	
Participant/StudyPartner/PI Blindedness Questionnaire								X	
Informed Consent (Subject)	X	R							
Informed Consent (Study Partner)	X	R[a]	R[e]	R[e]	R[e]	R[e]	R[e]	R[e]	
Assign Subject ID/ Unique Site ID number	X								
Inclusion/Exclusion Criteria	X	R							
Screening Demographics (Subject)	X								
Screening Demographics (Study Partner)	X	R[a]	R[e]	R[e]	R[e]	R[e]	R[e]	R[e]	
Medical & Surgical Historv	X	R							
Review methods of birth control with men and women	X	X							
Complete Phvsical Exam, including neurological	X							X	
Height, Weight (wt)	X				X(wt)			X(wt)	
Orthostatic Vital Signs (HR, BP), and Body Temperature	X	X[b]	X		X	X[b]	X	X	
Resting 12-Lead EKG	X	X	X		X	X	X	X	
Safety Labs (Hematology, Chemistry, Urinalysis, Study Drug Spot Check)	X	X[h]	X[g]		X[g]	X	X	X[g]	
Pregnancy Test (females only)	X(s)	X(u)			X (s)			X(s)	
Urine Drug Screen	X								
Blood Collections for CAGn Genotyping and storage		X	R[f]		R[f]	R[f]	R[f]	R[f]	
Randomization Assignment		X							
Dispense Study drug		X	X		X	X	X		
Instruct Subject and Study Partner about Study Dmg		X	X		X	X	X	X[c]	
Administer Study Drug on Site		X				X	(X)[d]		

Supplementary Table S2. Adverse Events by System Organ Class and Treatment Group

Column 1	2	3	4	5	6	7	8	9	10	11	12	13
	Group											
	120 mg N = 36			Placebo N = 36			160 mg N = 34			RR (95% CI)		
System Organ Class	# (%) of Subjects	# of Events	Rate	# (%) of Subjects	# of Events	Rate	# (%) of Subjects	# of Events	Rate	Placebo vs 120 mg	Placebo vs 160 mg	120 mg vs 160 mg
**Blood**												
Lymph node pain	1 (2.8%)	1	.009	0 (0.0%)	0	.000	0 (0.0%)	0	.000	N/A	N/A	N/A
**Cardiac**												
Angina pectoris	1 (2.8%)	1	.009	0 (0.0%)	0	.000	0 (0.0%)	0	.000	N/A	N/A	N/A
Atrioventricular block first degree	0 (0.0%)	0	.000	1 (2.8%)	1	.009	0 (0.0%)	0	.000	N/A	N/A	N/A
Bundle branch block left	0 (0.0%)	0	.000	1 (2.8%)	1	.009	0 (0.0%)	0	.000	N/A	N/A	N/A
Palpitations	0 (0.0%)	0	.000	0 (0.0%)	0	.000	1 (2.9%)	1	.009	N/A	N/A	N/A
**Gastrointestinal**												
Abdominal distension	1 (2.8%)	1	.009	0 (0.0%)	0	.000	0 (0.0%)	0	.000	N/A	N/A	N/A
Abdominal pain	1 (2.8%)	1	.009	0 (0.0%)	0	.000	0 (0.0%)	0	.000	N/A	N/A	N/A
Abdominal pain lower	0 (0.0%)	0	.000	1 (2.8%)	1	.009	0 (0.0%)	0	.000	N/A	N/A	N/A
Abdominal pain upper	1 (2.8%)	1	.009	1 (2.8%)	1	.009	3 (8.8%)	3	.026	1.00 (0.07, 15.38)	0.31 (0.03, 2.88)	0.31 (0.03, 2.88)
Constipation	0 (0.0%)	0	.000	1 (2.8%)	1	.009	0 (0.0%)	0	.000	N/A	N/A	N/A
Diarrhoea	1 (2.8%)	1	.009	0 (0.0%)	0	.000	1 (2.9%)	1	.009	N/A	N/A	0.94 (0.06, 14.51)
Dyspepsia	3 (8.3%)	3	.027	0 (0.0%)	0	.000	0 (0.0%)	0	.000	N/A	N/A	N/A
Gastrooesophageal reflux disease	0 (0.0%)	0	.000	0 (0.0%)	0	.000	1 (2.9%)	1	.009	N/A	N/A	N/A
Nausea	3 (8.3%)	4	.036	0 (0.0%)	0	.000	5 (14.7%)	5	.043	N/A	N/A	0.57 (0.15, 2.19)
Tongue disorder	1 (2.8%)	1	.009	0 (0.0%)	0	.000	0 (0.0%)	0	.000	N/A	N/A	N/A
Toothache	0 (0.0%)	0	.000	1 (2.8%)	1	.009	0 (0.0%)	0	.000	N/A	N/A	N/A
Vomiting	1 (2.8%)	1	.009	0 (0.0%)	0	.000	2 (5.9%)	4	.035	N/A	N/A	0.47 (0.04, 4.97)
**General**												
Crying	0 (0.0%)	0	.000	0 (0.0%)	0	.000	1 (2.9%)	2	.017	N/A	N/A	N/A
Fatigue	2 (5.6%)	2	.018	0 (0.0%)	0	.000	4 (11.8%)	4	.035	N/A	N/A	0.47 (0.09, 2.41)
Gait disturbance	0 (0.0%)	0	.000	1 (2.8%)	1	.009	1 (2.9%)	1	.009	N/A	0.94 (0.06, 14.51)	N/A
Influenza like illness	1 (2.8%)	1	.009	0 (0.0%)	0	.000	0 (0.0%)	0	.000	N/A	N/A	N/A
Pyrexia	0 (0.0%)	0	.000	1 (2.8%)	1	.009	0 (0.0%)	0	.000	N/A	N/A	N/A
**Infections**												
Bronchitis	1 (2.8%)	1	.009	0 (0.0%)	0	.000	0 (0.0%)	0	.000	N/A	N/A	N/A
Conjunctivitis	0 (0.0%)	0	.000	1 (2.8%)	1	.009	0 (0.0%)	0	.000	N/A	N/A	N/A
Cystitis	0 (0.0%)	0	.000	0 (0.0%)	0	.000	1 (2.9%)	1	.009	N/A	N/A	N/A
Influenza	0 (0.0%)	0	.000	2 (5.6%)	2	.018	0 (0.0%)	0	.000	N/A	N/A	N/A
Nasopharyngitis	1 (2.8%)	1	.009	0 (0.0%)	0	.000	0 (0.0%)	0	.000	N/A	N/A	N/A
Pharyngitis streptococcal	0 (0.0%)	0	.000	1 (2.8%)	1	.009	0 (0.0%)	0	.000	N/A	N/A	N/A
Pyelonephritis	1 (2.8%)	1	.009	0 (0.0%)	0	.000	0 (0.0%)	0	.000	N/A	N/A	N/A
Sinusitis	0 (0.0%)	0	.000	1 (2.8%)	1	.009	1 (2.9%)	1	.009	N/A	0.94 (0.06, 14.51)	N/A
Staphylococcal skin infection	0 (0.0%)	0	.000	1 (2.8%)	1	.009	0 (0.0%)	0	.000	N/A	N/A	N/A
Upper respiratory tract infection	1 (2.8%)	1	.009	3 (8.3%)	3	.027	2 (5.9%)	2	.017	0.33 (0.04, 3.06)	1.42 (0.25, 7.96)	0.47 (0.04, 4.97)
Urinary tract infection	2 (5.6%)	3	.027	1 (2.8%)	2	.018	0 (0.0%)	0	.000	2.00 (0.19, 21.09)	N/A	N/A
**Injury, Poisoning, and Procedural Comp.**												
Ankle fracture	1 (2.8%)	1	.009	0 (0.0%)	0	.000	0 (0.0%)	0	.000	N/A	N/A	N/A
Contusion	1 (2.8%)	1	.009	0 (0.0%)	0	.000	0 (0.0%)	0	.000	N/A	N/A	N/A
Fall	3 (8.3%)	3	.027	1 (2.8%)	1	.009	3 (8.8%)	6	.052	3.00 (0.33, 27.50)	0.31 (0.03, 2.88)	0.94 (0.20, 4.36)

Joint injury	0 (0.0%)	0	.000	1 (2.8%)	1	.009	0 (0.0%)	0	.000	N/A	N/A	N/A
Ligament sprain	0 (0.0%)	0	.000	1 (2.8%)	1	.009	0 (0.0%)	0	.000	N/A	N/A	N/A
Limb injury	0 (0.0%)	0	.000	1 (2.8%)	1	.009	0 (0.0%)	0	.000	N/A	N/A	N/A
Muscle strain	0 (0.0%)	0	.000	0 (0.0%)	0	.000	1 (2.9%)	1	.009	N/A	N/A	N/A
Post concussion syndrome	1 (2.8%)	1	.009	0 (0.0%)	0	.000	0 (0.0%)	0	.000	N/A	N/A	N/A
Tooth fracture	0 (0.0%)	0	.000	1 (2.8%)	1	.009	0 (0.0%)	0	.000	N/A	N/A	N/A
**Investigations**												
Alanine aminotransferase increased	0 (0.0%)	0	.000	0 (0.0%)	0	.000	2 (5.9%)	2	.017	N/A	N/A	N/A
Blood bilirubin increased	1 (2.8%)	1	.009	0 (0.0%)	0	.000	0 (0.0%)	0	.000	N/A	N/A	N/A
Blood creatinine increased	1 (2.8%)	1	.009	2 (5.6%)	2	.018	0 (0.0%)	0	.000	0.50 (0.05, 5.27)	N/A	N/A
Electrocardiogram QT prolonged	0 (0.0%)	0	.000	1 (2.8%)	1	.009	1 (2.9%)	1	.009	N/A	0.94 (0.06, 14.51)	N/A
Electrocardiogram T wave abnormal	1 (2.8%)	1	.009	0 (0.0%)	0	.000	0 (0.0%)	0	.000	N/A	N/A	N/A
Electrocardiogram abnormal	0 (0.0%)	0	.000	1 (2.8%)	1	.009	0 (0.0%)	0	.000	N/A	N/A	N/A
Eosinophil count increased	0 (0.0%)	0	.000	0 (0.0%)	0	.000	1 (2.9%)	1	.009	N/A	N/A	N/A
Glucose urine	1 (2.8%)	1	.009	0 (0.0%)	0	.000	0 (0.0%)	0	.000	N/A	N/A	N/A
Glucose urine present	0 (0.0%)	0	.000	1 (2.8%)	1	.009	0 (0.0%)	0	.000	N/A	N/A	N/A
Hepatic enzyme increased	3 (8.3%)	3	.027	1 (2.8%)	1	.009	1 (2.9%)	1	.009	3.00 (0.33, 27.50)	0.94 (0.06, 14.51)	2.83 (0.31, 25.94)
Platelet count decreased	0 (0.0%)	0	.000	1 (2.8%)	1	.009	1 (2.9%)	1	.009	N/A	0.94 (0.06, 14.51)	N/A
Urine leukocyte esterase positive	0 (0.0%)	0	.000	0 (0.0%)	0	.000	2 (5.9%)	2	.017	N/A	N/A	N/A
Weight increased	1 (2.8%)	1	.009	1 (2.8%)	1	.009	1 (2.9%)	1	.009	1.00 (0.07, 15.38)	0.94 (0.06, 14.51)	0.94 (0.06, 14.51)
**Metabolic**												
Dehydration	1 (2.8%)	1	.009	1 (2.8%)	1	.009	0 (0.0%)	0	.000	1.00 (0.07, 15.38)	N/A	N/A
**Musculoskeletal**												
Arthralgia	1 (2.8%)	1	.009	1 (2.8%)	1	.009	2 (5.9%)	2	.017	1.00 (0.07, 15.38)	0.47 (0.04, 4.97)	0.47 (0.04, 4.97)
Back pain	0 (0.0%)	0	.000	0 (0.0%)	0	.000	1 (2.9%)	1	.009	N/A	N/A	N/A
Costochondritis	1 (2.8%)	1	.009	0 (0.0%)	0	.000	0 (0.0%)	0	.000	N/A	N/A	N/A
Muscular weakness	1 (2.8%)	1	.009	0 (0.0%)	0	.000	0 (0.0%)	0	.000	N/A	N/A	N/A
Musculoskeletal chest pain	1 (2.8%)	1	.009	0 (0.0%)	0	.000	0 (0.0%)	0	.000	N/A	N/A	N/A
Neck pain	1 (2.8%)	1	.009	0 (0.0%)	0	.000	0 (0.0%)	0	.000	N/A	N/A	N/A
Osteoporosis	0 (0.0%)	0	.000	0 (0.0%)	0	.000	1 (2.9%)	1	.009	N/A	N/A	N/A
Pain in extremity	0 (0.0%)	0	.000	0 (0.0%)	0	.000	2 (5.9%)	2	.017	N/A	N/A	N/A
**Neoplasms**												
Renal neoplasm	1 (2.8%)	1	.009	0 (0.0%)	0	.000	0 (0.0%)	0	.000	N/A	N/A	N/A
Squamous cell carcinoma	0 (0.0%)	0	.000	0 (0.0%)	0	.000	1 (2.9%)	1	.009	N/A	N/A	N/A
**Nervous System**												
Anosmia	1 (2.8%)	1	.009	0 (0.0%)	0	.000	0 (0.0%)	0	.000	N/A	N/A	N/A
Chorea	0 (0.0%)	0	.000	1 (2.8%)	1	.009	0 (0.0%)	0	.000	N/A	N/A	N/A
Dizziness	2 (5.6%)	2	.018	1 (2.8%)	1	.009	1 (2.9%)	1	.009	2.00 (0.19, 21.09)	0.94 (0.06, 14.51)	1.89 (0.18, 19.89)
Dysarthria	0 (0.0%)	0	.000	1 (2.8%)	1	.009	0 (0.0%)	0	.000	N/A	N/A	N/A
Headache	3 (8.3%)	3	.027	1 (2.8%)	1	.009	3 (8.8%)	3	.026	3.00 (0.33, 27.50)	0.31 (0.03, 2.88)	0.94 (0.20, 4.36)
Hypersomnia	0 (0.0%)	0	.000	1 (2.8%)	1	.009	0 (0.0%)	0	.000	N/A	N/A	N/A
Lethargy	1 (2.8%)	1	.009	0 (0.0%)	0	.000	0 (0.0%)	0	.000	N/A	N/A	N/A
Paraesthesia	0 (0.0%)	0	.000	0 (0.0%)	0	.000	1 (2.9%)	1	.009	N/A	N/A	N/A
Somnolence	0 (0.0%)	0	.000	4 (11.1%)	4	.035	1 (2.9%)	1	.009	N/A	3.78 (0.44, 32.13)	N/A

**Psychiatric**												
Abnormal dreams	0 (0.0%)	0	.000	3 (8.3%)	3	.027	0 (0.0%)	0	.000	N/A	N/A	N/A
Aggression	0 (0.0%)	0	.000	1 (2.8%)	1	.009	1 (2.9%)	1	.009	N/A	0.94 (0.06, 14.51)	N/A
Agitation	1 (2.8%)	1	.009	0 (0.0%)	0	.000	0 (0.0%)	0	.000	N/A	N/A	N/A
Anxiety	1 (2.8%)	1	.009	0 (0.0%)	0	.000	0 (0.0%)	0	.000	N/A	N/A	N/A
Apathy	0 (0.0%)	0	.000	0 (0.0%)	0	.000	1 (2.9%)	1	.009	N/A	N/A	N/A
Depressed mood	2 (5.6%)	2	.018	0 (0.0%)	0	.000	1 (2.9%)	1	.009	N/A	N/A	1.89 (0.18, 19.89)
Hallucination, auditory	0 (0.0%)	0	.000	1 (2.8%)	1	.009	0 (0.0%)	0	.000	N/A	N/A	N/A
Hypervigilance	1 (2.8%)	1	.009	0 (0.0%)	0	.000	0 (0.0%)	0	.000	N/A	N/A	N/A
Indifference	0 (0.0%)	0	.000	0 (0.0%)	0	.000	1 (2.9%)	1	.009	N/A	N/A	N/A
Insomnia	2 (5.6%)	2	.018	0 (0.0%)	0	.000	1 (2.9%)	1	.009	N/A	N/A	1.89 (0.18, 19.89)
Intentional self-injury	1 (2.8%)	1	.009	0 (0.0%)	0	.000	0 (0.0%)	0	.000	N/A	N/A	N/A
Irritability	1 (2.8%)	1	.009	1 (2.8%)	1	.009	1 (2.9%)	1	.009	1.00 (0.07, 15.38)	0.94 (0.06, 14.51)	0.94 (0.06, 14.51)
Psychotic disorder	1 (2.8%)	1	.009	0 (0.0%)	0	.000	0 (0.0%)	0	.000	N/A	N/A	N/A
Sleep disorder	0 (0.0%)	0	.000	0 (0.0%)	0	.000	1 (2.9%)	1	.009	N/A	N/A	N/A
Suicidal ideation	1 (2.8%)	1	.009	0 (0.0%)	0	.000	1 (2.9%)	1	.009	N/A	N/A	0.94 (0.06, 14.51)
Suicide attempt	0 (0.0%)	0	.000	0 (0.0%)	0	.000	1 (2.9%)	1	.009	N/A	N/A	N/A
**Renal**												
Dysuria	0 (0.0%)	0	.000	0 (0.0%)	0	.000	1 (2.9%)	1	.009	N/A	N/A	N/A
Proteinuria	1 (2.8%)	1	.009	1 (2.8%)	1	.009	0 (0.0%)	0	.000	1.00 (0.07, 15.38)	N/A	N/A
Renal cyst	1 (2.8%)	1	.009	0 (0.0%)	0	.000	0 (0.0%)	0	.000	N/A	N/A	N/A
Urinary hesitation	0 (0.0%)	0	.000	0 (0.0%)	0	.000	1 (2.9%)	1	.009	N/A	N/A	N/A
**Reproductive**												
Dysmenorrhoea	1 (2.8%)	1	.009	0 (0.0%)	0	.000	0 (0.0%)	0	.000	N/A	N/A	N/A
**Respiratory**												
Asthma	0 (0.0%)	0	.000	0 (0.0%)	0	.000	1 (2.9%)	1	.009	N/A	N/A	N/A
Cough	2 (5.6%)	2	.018	1 (2.8%)	1	.009	0 (0.0%)	0	.000	2.00 (0.19, 21.09)	N/A	N/A
Dry throat	1 (2.8%)	1	.009	0 (0.0%)	0	.000	0 (0.0%)	0	.000	N/A	N/A	N/A
Dyspnoea	0 (0.0%)	0	.000	0 (0.0%)	0	.000	1 (2.9%)	1	.009	N/A	N/A	N/A
Nasal congestion	1 (2.8%)	1	.009	0 (0.0%)	0	.000	0 (0.0%)	0	.000	N/A	N/A	N/A
Oropharyngeal pain	0 (0.0%)	0	.000	0 (0.0%)	0	.000	1 (2.9%)	1	.009	N/A	N/A	N/A
Rhinitis allergic	1 (2.8%)	1	.009	0 (0.0%)	0	.000	0 (0.0%)	0	.000	N/A	N/A	N/A
Sleep apnoea syndrome	0 (0.0%)	0	.000	0 (0.0%)	0	.000	1 (2.9%)	1	.009	N/A	N/A	N/A
**Skin**												
Dermatitis acneiform	1 (2.8%)	1	.009	0 (0.0%)	0	.000	0 (0.0%)	0	.000	N/A	N/A	N/A
Hyperhidrosis	1 (2.8%)	1	.009	0 (0.0%)	0	.000	0 (0.0%)	0	.000	N/A	N/A	N/A
Night sweats	0 (0.0%)	0	.000	1 (2.8%)	1	.009	0 (0.0%)	0	.000	N/A	N/A	N/A
Precancerous skin lesion	0 (0.0%)	0	.000	0 (0.0%)	0	.000	1 (2.9%)	1	.009	N/A	N/A	N/A
**Vascular**												
Hot flush	0 (0.0%)	0	.000	0 (0.0%)	0	.000	1 (2.9%)	1	.009	N/A	N/A	N/A
Hypertension	0 (0.0%)	0	.000	0 (0.0%)	0	.000	1 (2.9%)	1	.009	N/A	N/A	N/A
Hypotension	1 (2.8%)	1	.009	0 (0.0%)	0	.000	0 (0.0%)	0	.000	N/A	N/A	N/A
**Total**	**29 (80.6%)**	**75**	**.676**	**26 (72.2%)**	**52**	**.460**	**30 (88.2%)**	**73**	**.635**	**1.12 (0.86, 1.44)**	**0.82 (0.65, 1.04)**	**0.91 (0.75, 1.12)**

Column Definitions:
1: System Organ Class of AE; bold terms are the System Organ Class of AE
2,5,8: Number and percentage of subjects who have ever had AE
3,6,9: Total number of AEs; includes multiple AEs of same type per subject
4,7,10: Number of AEs per 30 days
11: Relative Risk (120 mg relative to Placebo) and 95% confidence interval
12: Relative Risk (Placebo relative to 160 mg) and 95% confidence interval
13: Relative Risk (120 mg relative to 160 mg) and 95% confidence interval

Supplementary Figure S1. The power analysis is shown for the placebo single-arm sample size (n_P) as a function of the difference of the group completer proportions ($\pi_P - \pi_T$) and the marginal placebo proportion (π_P). Paneling is by α-level, with 80% power. In the left-hand panel, the upper red line shows that conducting two tests each at α = .025 and 80% power requires approximately 36 participants per group for the smallest proposed control proportion ($\pi_P = .80$) and the largest proportion difference ($\pi_P - \pi_T = .20$). Therefore, the projected total sample size of $N = 3n_P =$ was 108, and a sample size of $2n_P = 72$ for each comparison (120mg vs placebo, 160mg vs placebo). The right panel shows the n's for conducting two tests at α = .05.

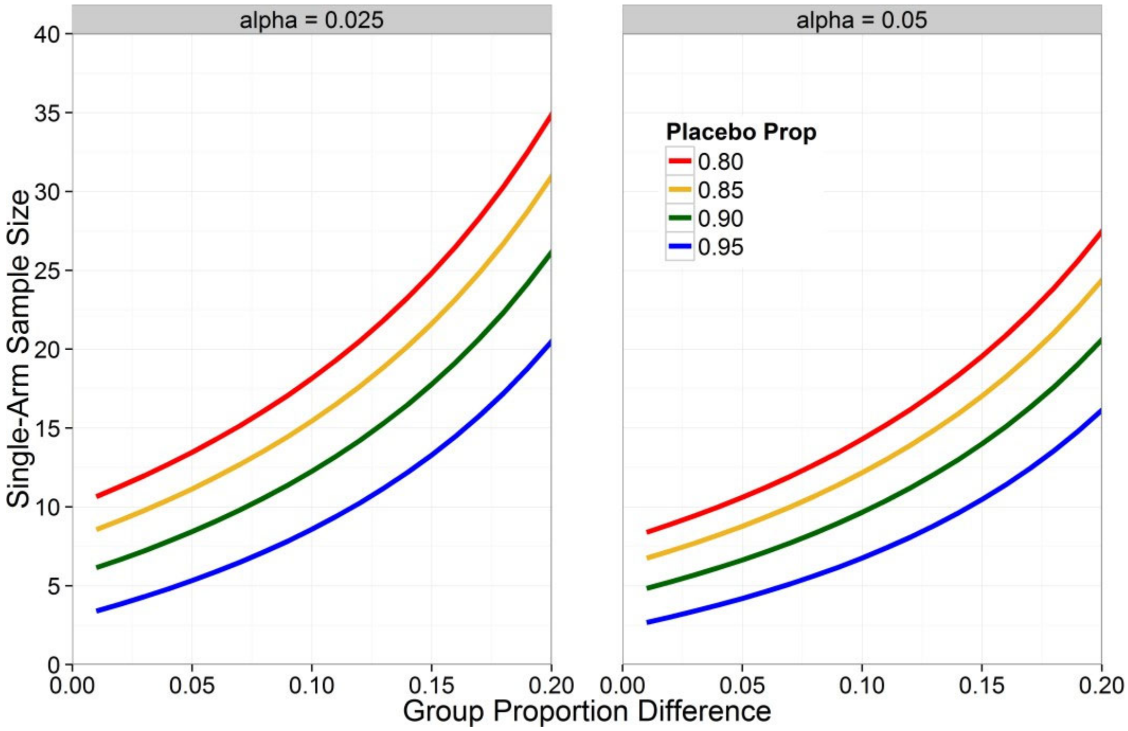

Supplement: Supplementary file 1 [file jcm-09-03682-s001.pdf]
